# Biological functions of tsRNAs and research advances in human disease

**DOI:** 10.1016/j.bbrep.2025.102438

**Published:** 2026-01-06

**Authors:** Yanting Jiang, Shuyou Liu, Guolin Li, Chengcheng Tang, Yangyang Zhang, Junjie Li, Zhirong Tan, Shimeng Zhang, Zhiyou Chen, Shulong Li

**Affiliations:** a Shenzhen Qianhai Shekou Free Trade Zone Hospital, Shenzhen, China; b Second Clinical Medical College, Guangdong Medical University, Dongguan, China

**Keywords:** tsRNA, Non-coding RNA, Biological function, Disease

## Abstract

tsRNAs (tRNA-derived small RNAs) are a class of noncoding small RNAs generated by nuclease-specific cleavage of mature tRNAs or their precursors. Since their functional discovery in 2009, tsRNAs have emerged as a research hotspot in molecular biology and medicine because of their unique generation mechanism, tissue-specific expression patterns, and diverse regulatory functions. Compared with traditional non-coding RNAs (such as miRNAs and lncRNAs), tsRNAs exhibit distinct biological activities in stress responses, translational regulation, and epigenetic modifications and are closely associated with the onset and progression of various human diseases. This systematic review covers the discovery history, classification characteristics, primary biological functions, and action mechanisms of tsRNAs in major diseases, including respiratory disorders, neuropsychiatric conditions, cardiovascular diseases, and tumors. It further explores the clinical translational potential of tsRNAs as disease biomarkers and therapeutic targets, identifies current challenges, and outlines future research directions. Major knowledge gaps highlighted include the lack of standardized detection methods for tsRNA subtypes, unclear specificity of their molecular targets in pathological processes, and limited validation in large clinical cohorts; key challenges involve inefficient delivery of tsRNA-based therapeutics and insufficient exploration of cross-species conservation. The review aims to provide a comprehensive reference for in-depth studies in the field of tsRNA.

## Highlights

• TsRNAs (emerging noncoding RNAs, from tRNA cleavage, tRFs/tiRNAs) regulate physiology/pathology, link to tumors, have potential but challenges; future tech/multiomics aid disease research.

Non-coding RNAs (ncRNAs) refer to RNA molecules within the genomic transcriptome that do not encode proteins, and have emerged as a research hotspot in the life sciences in recent years. Among these, tRNA-derived small RNAs (tsRNAs) were long considered nonfunctional byproducts of tRNA degradation. This perception changed in 2009 when Fu et al. successfully isolated tRNA fragments derived from tRNA cleavage within the anticodon loop. These fragments were found to be widely distributed across various cell lines and mouse tissues [1]. Typically ranging from 18 to 40 nucleotides (nt) in length, tsRNAs are generated through cleavage of mature tRNAs or tRNA precursors by enzymes such as Angiogenin, Dicer, RNase Z, and RNase P. They are detected in multiple human bodily fluids [2,3]. Based on their generation mechanisms and structural characteristics, tsRNAs can be categorized into two major classes: tRFs (tRNA-derived fragments) and tiRNAs (tRNA-derived stress-induced small RNAs) [4,5].

Compared to other non-coding RNAs like miRNAs, tsRNAs possess the following unique advantages: (1) High stability: Due to their rich modifications (e.g., m^1^A, pseudouridine) and short fragment length, they exhibit strong resistance to nucleases [6,7]; (2) Stress response specificity: Rapidly upregulated under conditions like hypoxia and oxidative stress, serving as key regulators of cellular stress responses [6,8]; (3) Significant tissue specificity: TsRNA expression profiles exhibit marked differences across tissues, offering potential for precise disease diagnosis [7,8].

To better contextualize the distinct biological relevance of tsRNAs, we briefly compare them with two other emerging small RNA fragments: piwi-interacting RNAs (piRNAs) and snoRNA-derived RNAs (sdRNAs). In terms of biogenesis, piRNAs are primarily derived from long intergenic repeat regions and mature via PIWI protein-mediated processing [9], while sdRNAs originate from small nucleolar RNAs (snoRNAs) through endonucleolytic cleavage [10]; they both differ from tsRNAs' tRNA-derived origin. Functionally, piRNAs are predominantly restricted to germ cells, where they silence transposons to maintain genomic stability [9,11]; sdRNAs mainly regulate ribosomal RNA modification or pre-mRNA splicing [10]. In contrast, tsRNAs are broadly expressed across somatic and germ cells, with diverse roles in stress responses, translational control, and epigenetic regulation—filling a unique niche in integrating cellular physiology and environmental adaptation [12]. Disease relevance further distinguishes tsRNAs: while piRNAs are linked mainly to germline tumors and sdRNAs to rare ribosomopathy-related disorders [13,14], tsRNAs are associated with a wide spectrum of common diseases (e.g., tumors, neurodegenerative conditions, metabolic disorders), underscoring their broader clinical translational potential [7,15].

Currently, research on tsRNAs has expanded from fundamental mechanisms to clinical applications. The elucidation of their abnormal expression patterns and regulatory networks in various diseases has opened new avenues for early diagnosis, prognosis assessment, and targeted therapy. This article systematically reviews research progress in the tsRNA**s'** field, focusing on their biological functions and the molecular mechanisms linking them to disease.

## 1 Classification characteristics and generation mechanisms of tsRNAs

**Fig. 1:**
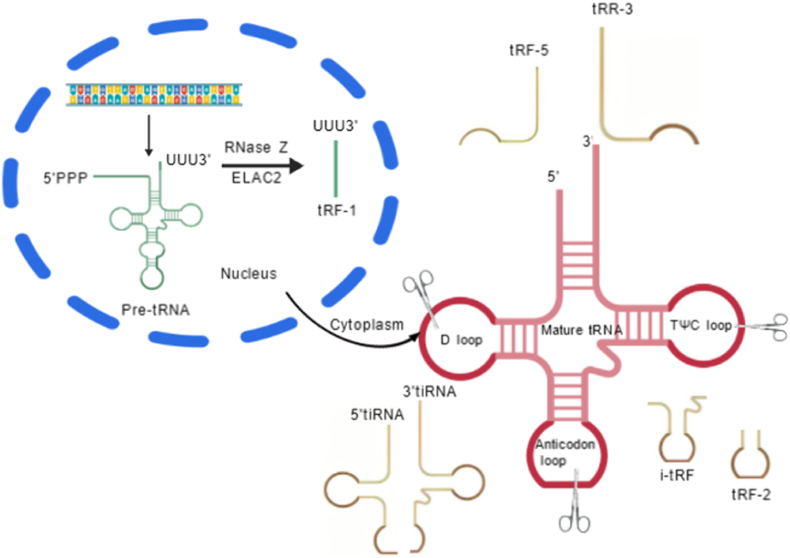
Classification characteristics of tsRNA**s**.

Based on generation mechanisms and structural features, tsRNAs are primarily categorized into two major types: tRNA-derived fragments (tRFs) and tRNA-derived stress-induced small RNAs (tiRNAs) (see Fig. 1). These two types exhibit distinct functional roles: tRFs are widely involved in fundamental physiological regulation, while tiRNAs tend to function under stress conditions [6,7,16].

### 1.1 tRNA-derived fragments (tRFs)

tRNA-derived fragments (tRFs) constitute a subtype of tsRNAs generated by specific nuclease cleavage of mature tRNAs or their precursors. Their classification primarily relies on differences in cleavage sites, yielding the following core subtypes:

1) tRF-5: Originating from the 5′ end region of tRNA, generated by Dicer or angiogenin (ANG)-mediated cleavage of the D loop or the region between the D loop and the anticodon loop. Based on subtle differences in cleavage sites, these are further subdivided into tRF-5a, tRF-5b, and tRF-5c subtypes. Their lengths typically range from 14 to 30 nt, and some subtypes can participate in the RNA interference pathway by binding to Argonaute proteins [17].
2) tRF-3: In contrast, tRF-3 originates from the 3′ end region of tRNA. After cleavage, it retains the conserved CCA tail sequence inherent to tRNA. Based on cleavage position, it is subdivided into tRF-3a and tRF-3b. This subtype typically spans 18–22 nt and regulates gene expression by complementary binding to the 3′ untranslated region (3′UTR) of mRNA [18].
3) tRF-1: As the sole subtype originating from precursor tRNA (pre-tRNA), tRF-1 is processed by RNase Z/ELAC2 cleaving the 3′ end of pre-tRNA, resulting in lengths ranging from 16 to 48 nt. Due to its characteristic polyuridine tract (polyU tract) at the 3′ end, it is also known as 3′U-tRF. This sequence feature enables it to readily bind to adenine-rich regions of mRNA, thereby affecting mRNA stability or translation efficiency [19].

Furthermore, recent studies have expanded knowledge on tRF-2 and i-tRF (internal tRF) subtypes. Though not included in traditional classification systems, these have been demonstrated to participate in physiological processes such as cell cycle regulation, enriching the tRF family [16,20].

The varied cleavage sites (5′ end, 3′ end, pre-tRNA) and structural signatures (e.g., CCA tail of tRF-3, polyU tract of tRF-1) of tRF subtypes are not arbitrary; instead, these features directly shape the molecular interfaces that allow tRFs to interact with specific cellular targets (e.g., mRNA 3′UTR, ribosomal subunits), laying the groundwork for their specialized roles in basal physiological regulation (e.g., constitutive translation control).

### 1.2 tRNA-derived stress-induced small RNAs (tiRNAs)

tRNA-derived stress-induced small RNAs (tiRNAs) constitute a class of tsRNAs specifically generated under stress conditions (e.g., heat shock, oxidative stress, hypoxia), characterized by stress-signal-dependent inducible expression.

tiRNAs are primarily generated by ANG cleavage of mature tRNAs, yielding tRNA half-molecules of characteristic lengths. They are classified into two main types:

1) 5′tiRNA: Approximately 30–35 nt in length, containing the amino acid acceptor arm and part of the anticodon loop sequence at the 5′ end of tRNA. It forms a stable stem-loop secondary structure, which is the key foundation for its participation in stress particle assembly [21,22]^.^ ;
2) 3′tiRNA: Approximately 40–45 nt in length, containing the anticodon loop and CCA tail at the 3′ end of tRNA. It can form transient complementary base pairing with mRNA codons, thereby regulating the translation process [21,23].

Notably, recent studies indicate that tiRNA biogenesis relies not only on ANG but also involves multiple nucleases including RNase I, endonuclease V, Schlafen13 (SFLN13), and RNase T2/L, collectively forming an intricate regulatory network [[24], [25], [26], [27], [28]]. This discovery suggests that the tiRNA generation mechanism is far more complex than traditionally understood, with its expression regulation potentially closely linked to stress-type specificity. This provides a new perspective for deepening our understanding of the molecular networks underlying cellular stress responses.

Collectively, the mechanistic diversity of tsRNAs is reflected in two key forms: tRFs undergo site-specific, constitutive cleavage, while tiRNAs rely on stress-inducible, multi-enzyme processing. This diversity establishes a “structure-function blueprint”: each subtype's generation pathway encodes unique structural and regulatory properties, which in turn dictate their distinct biological roles.

## 2 Biological functions of tsRNA

Building on the diverse biogenesis mechanisms outlined in Section 1 (e.g., tRFs' cleavage-site specificity, tiRNAs' stress-dependent generation), tsRNAs exhibit biological functions that directly align with their mechanistic origins. Specifically, structural features shaped by their biogenesis, including tRF-3's CCA tail and 5'tiRNA's G-quadruplex, enable interactions with distinct cellular components (mRNAs, ribosomes, epigenetic enzymes), supporting regulation of core biological processes, starting with protein translation.

**Fig. 2:**
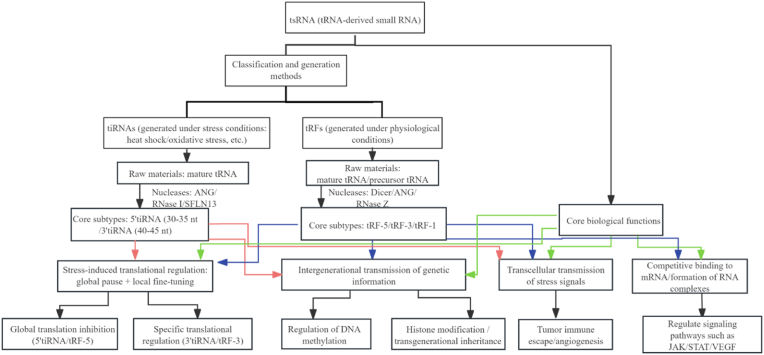
Classification, synthesis pathways of tsRNAs, and a schematic illustration of their core biological functions.

This focus on translation regulation, which is rooted in their biogenesis, highlights a broader pattern of tsRNA function: as tRNA-derived small RNAs, they also possess notable cellular specificity, with roles spanning both fundamental physiological regulation (e.g., maintaining basal translation) and pathophysiological responses (e.g., stress adaptation, disease progression). Key functional directions include regulating protein translation, mediating epigenetic modifications, acting as intercellular signaling molecules, and cross-regulating with other non-coding RNAs (see Fig. 2).

### 2.1 Regulation of protein translation

tsRNA regulation of protein translation is bidirectional and specific, with different subtypes influencing translation initiation, elongation, or termination through distinct mechanisms:

#### 2.1.1 Fundamental regulation of translation by tRFs

tRFs primarily participate in fundamental translation regulation under physiological conditions through mechanisms including:

1) Translation initiation inhibition: tRF-3 can bind complementarily to the 3′ untranslated region (3′UTR) of mRNA, hindering ribosomal recognition of the mRNA and thereby downregulating the translation efficiency of target genes [22,29]; Certain tRF-5s (e.g., tRF-5a) bind to the ribosomal small subunit (40S) via a conserved “GG” dinucleotide motif or directly interact with the translation initiation factor eIF4G, inhibiting assembly of the translation initiation complex to achieve global translation repression [16,30,31].
2) Translation enhancement: Some tRFs indirectly enhance translation by promoting the stability of specific mRNAs. For example, tRF-LeuCAG binds to ribosomal proteins RPS28 and RPS15, thereby increasing the translation efficiency of target mRNAs [32,33].

#### 2.1.2 Stress-induced translational regulation by tiRNAs

tiRNAs primarily initiate translational regulation under stress conditions (e.g., oxidative stress, starvation), serving as a key mechanism for cellular adaptation to environmental pressures [[34], [35], [36], [37]]. This stress-induced translational regulation is vital for cellular adaptation and survival. Under stress, translational control initiated by tiRNAs operates not as a single mode but through a synergistic network formed by the functional differentiation of 5**′**tiRNAs and 3**′**tiRNAs: the former act as **“**throttles**”** via global suppression, reducing non-essential protein synthesis to conserve energy; while the latter enables “precision regulation” through specific targeting, prioritizing the synthesis of stress-related proteins or suppressing the expression of harmful proteins. This dual mechanism of **“**global pausing and local fine-tuning**”** ensures energy homeostasis for maintaining basal metabolism under extreme conditions while enhancing stress adaptability through selective translational control. Together, they constitute the core survival strategy for cells confronting adverse environments.

1) Global translation inhibition: 5′tiRNAs form stable stem-loop structures (particularly G-quadruplexes), binding to core stress granule proteins (e.g., G3BP1) to induce stress granule assembly. This pauses global protein synthesis, helping cells conserve energy under adverse conditions [38,39].
2) Specific translation elongation inhibition: 3′tiRNAs competitively bind the ribosomal A site by transiently complementary pairing with mRNA codons, preventing aminoacyl-tRNA entry and thus specifically inhibiting translation elongation [21,40].

### 2.2 Epigenetic regulatory functions

Epigenetic regulation dynamically modulates gene expression through mechanisms such as DNA methylation and histone modifications, playing a crucial role in cellular differentiation, stress responses, and disease development [[41], [42], [43]]. As key participants in epigenetic regulation, tsRNAs can interact with enzymes involved in epigenetic modifications to target specific gene regions, thereby regulating modification states and influencing gene expression. Their effects are primarily manifested at the levels of DNA methylation and histone modifications [44].

In DNA methylation regulation, tsRNAs interact with DNA methyltransferases, guiding them to specific gene regions to modulate methylation levels [45,46]. For instance, Wu et al. discovered that tiRNA-Gly-GCC indirectly downregulates DNMT3A protein levels, reducing PTEN promoter methylation, restoring PTEN expression, and inhibiting the PI3K/AKT pathway [47].

Regarding histone modifications, tsRNAs can influence the activity of histone methyltransferases (e.g., EZH2) and deacetylases (e.g., HDACs) [48]. Some tsRNAs also directly interact with histone-modifying enzymes, regulating their activity or localization to alter histone methylation/acetylation levels. For example, the hypoxia-responsive tRNA-Asp-GTC-3′tDR interacts with pseudouridine synthase (PUS7), preventing its pseudouridination modification of histone mRNA and thereby triggering RNA autophagy, demonstrating that the association between tsRNAs and histone modification exerts a renal protective effect. [49]. Furthermore, m5C is a common modification in tsRNAs, catalyzed by enzymes such as NOP2/Sun domain family member 2 (NSun2). NSun2 deficiency leads to reduced tRNA methylation, impairing tsRNA production and function [50].

tsRNAs may also participate in transgenerational information transfer: the expression profiles and modification states of tsRNAs in germ cells can be inherited between parents and offspring. For example, 5′-tiRNAs induced by starvation stress in nematode germ cells can be transmitted through the germline to offspring, enhancing their resistance to viral infection [51]; In mammals, a high-fat diet in paternal mice alters the expression profiles and m5C modification levels of tsRNAs (particularly tRF-3 and tRF-5 subtypes) in sperm. These tsRNAs enter the zygote post-fertilization and influence offspring metabolic phenotypes (e.g., obesity susceptibility) by regulating early embryonic metabolism-related gene expression [52]. These findings enrich our understanding of the epigenetic regulatory role of tsRNAs, suggesting their potential as therapeutic targets for disease treatment [53]. Future discoveries of additional mechanisms will provide new perspectives for understanding biological processes and disease pathogenesis.

### 2.3 As intercellular signaling molecules

tsRNAs **are selectively packaged into exosomes through sequence-specific recognition by RNA-binding proteins (RBPs), including sumoylated hnRNPA2B1 (recognizing GG dinucleotide motifs) and YBX1 (mediated by liquid-liquid phase separation)** [[54], [55], [56]]**.** They can be secreted via exosomes and taken up by target cells, serving as important intercellular signaling molecules [2,57]. In the tumor microenvironment, tumor cell-secreted exosomal tsRNAs, when taken up by fibroblasts, immune cells, and others, can alter the functions of these cells.

For example, tRF-3021a in colorectal cancer cell exosomes upregulates **A Disintegrin and Metalloproteinase Domain 10 (**ADAM10) protein expression, promoting the proteolytic cleavage of membrane **MHC class I chain-related gene A** (mMICA) into soluble MICA (sMICA). As a decoy ligand for the NK receptor group 2 member D (NKG2D) receptor on NK cells, sMICA reduces cytotoxicity, facilitates immune evasion, and thereby promotes tumor proliferation and metastasis [58]. Regarding anti-angiogenesis, tRNA-Cys-5-0007 expression is downregulated under angiogenic conditions. However, its overexpression in retinal endothelial cells reduces cell proliferation, sprouting, migration, and tubule formation by targeting **vascular endothelial growth factor A (**VEGFA) and TGF-β1. In diabetic, laser-induced Choroidal Neovascularization (CNV), and Oxygen-Induced Retinopathy (OIR) models, its overexpression also reduces ocular vascular leakage, inhibits angiogenesis, and alleviates inflammation [59]. Furthermore, tRF-1003 expression is significantly upregulated in multiple myeloma exosomes, where it induces angiogenesis by modulating the HIF1α/VEGF signaling pathway [60].

Evidently, as intercellular signaling molecules, tsRNAs are crucial for intercellular communication and overall functional coordination under both physiological and pathological conditions.

### 2.4 Cross-regulation with other non-coding RNAs

tsRNAs exhibit complex cross-regulatory relationships with other non-coding RNAs, such as miRNAs and lncRNAs. Through direct interactions or indirect regulatory mechanisms, they jointly influence cellular biological functions. In-depth exploration of these mechanisms aids in understanding the role of tsRNAs in disease and provides novel therapeutic strategies.

tsRNAs can modulate miRNA regulation by competitively binding target mRNAs [61]. For example, in chondrocytes, tRF-3003a binds to AGO2/GW182 proteins, competitively inhibiting other miRNAs from binding to this protein complex. This allows tRF-3003a to target the 3′UTR region of JAK3, blocking the JAK/STAT signaling pathway and downregulating IL-6 expression to alleviate inflammatory responses [62].

tsRNAs can also interact with lncRNAs to form RNA-RNA complexes, cooperatively recruiting to specific gene regions (e.g., promoters) to jointly regulate gene expression [63,64]. For instance, in cardiovascular diseases, the ginseng-derived tRF-hc83 binds to the lncRNA myocardial **infarction associated transcript (**MIAT) through base complementary pairing, inhibiting its interaction with VEGFA mRNA. This releases MIAT's suppression of VEGFA, thereby promoting vascular endothelial growth factor (VEGF) expression, improving myocardial angiogenesis, and reducing apoptosis [65]. This cross-regulation further enriches the complexity and precision of intracellular gene expression regulatory networks.

## 3 Research progress on tsRNAs in human diseases

Current research indicates that the disease-associated spectrum of tsRNAs spans multiple domains including tumors, neurological disorders, cardiovascular diseases, metabolic disorders, and infectious diseases. They exhibit distinct mechanisms of action and clinical significance across different disease systems. The following sections systematically review tsRNA research findings across various disease categories, elucidating their molecular mechanisms in disease pathogenesis and application potential, thereby providing references for future basic research and clinical translation.

### 3.1 Neoplastic diseases

Research on tsRNAs in oncology is the most systematic. Abnormal expression has been identified in multiple solid tumors and hematologic malignancies, where tsRNAs participate in carcinogenesis by regulating core biological processes such as cell proliferation, apoptosis, invasion/metastasis, and drug resistance. Some mechanisms have been validated in both clinical samples and animal models [66].

#### 3.1.1 Solid tumors

The role of tsRNAs in solid tumors exhibits significant tissue specificity. Their molecular mechanisms encompass multiple modes, including competitive binding to miRNAs, forming complexes with lncRNAs to regulate target gene transcription, and directly inhibiting the translation of tumor suppressor genes.

1) Digestive system tumors:

In hepatocellular carcinoma (HCC), hepatocellular carcinoma-effectively-associated tsRNA (HCETSR) derived from tRNA-Glu/TTC effectively suppresses malignant progression by regulating the SPTBN1/catenin axis, emerging as a promising prognostic biomarker and therapeutic target for HCC [67]. Another study revealed that tRF-3023 directly targets the mRNA of the cell cycle suppressor Cyclin-dependent kinase inhibitor 1A (CDKN1A, p21), accelerating the transition from G1 to S phase and thereby promoting malignant proliferation in hepatocellular carcinoma cells [68].

In colorectal cancer (CRC), tsRNA-GlyGCC is upregulated in CRC tissues and regulated by N(7)-methylguanosine tRNA modification. In vitro and in vivo experiments indicate that tsRNA-GlyGCC promotes CRC resistance to 5-fluorouracil by targeting Spleen-associated tyrosine kinase-binding protein (SPIB) to regulate the JAK1/STAT6 signaling pathway [69]; while tRF3008A suppresses proliferation and migration of CRC in vivo and in vitro by inhibiting endogenous Forkhead box protein K1 (FOXK1, a positive regulator of the Wnt/β-catenin pathway) [70].

In gastric cancer (GC), tRF-19-3L7L73JD effectively inhibits proliferation and migration of GC cells, induces apoptosis, and causes cell arrest in the G0/G1 phase, suggesting its potential inhibitory role in GC progression [71]. Conversely, tRF-23-Q99P9P9NDD binds to the 3′ untranslated region (UTR) site of acyl-CoA dehydrogenase short/branched-chain (ACADSB), affecting lipid metabolism and ferroptosis processes in GC, thereby promoting its progression [72]. Furthermore, tRF-Tyr directly binds to heterogeneous nuclear ribonucleoprotein D (hnRNPD) protein and competitively inhibits hnRNPD's interaction with the c-Myc 3′UTR, thereby regulating the c-Myc/Bcl2/Bax signaling pathway and ultimately suppressing GC progression [73].

2) Neurological tumors:

In gliomas, tRNA methyltransferase 10 homologue A (TRMT10A) expression is significantly downregulated in glioma cells, leading to reduced m1G9 modification of tRNA-argcct. This decreases tRNA stability and upregulates tRF-22 expression levels. tRF-22 negatively regulates **MAX dimerization protein 1** (MXD1) expression by binding to its 3′UTR, thereby weakening MXD1's transcriptional suppression of hypoxia-inducible factor 1 alpha (HIF1A) and promoting glioma vascular mimicry (VM) formation [74]. Additionally, significantly reduced expression of ts-26 and tRFdb-3012a/b derived from tRNA-Leu-CAA correlates with poorer survival in low-expressing patients. These molecules, associated with mutations like isocitrate dehydrogenase (IDH), may target RNA-binding motif protein 43 (RBM43) and homeobox A13 (HOXA13), whose overexpression is strongly linked to poor prognosis [75].

3) Respiratory tumors:

In non-small cell lung cancer (NSCLC), AS-tDR-007333 is a novel oncogenic tRF that activates **mediator of RNA polymerase II transcription subunit 29 (**MED29) through two distinct mechanisms to promote NSCLC cell malignancy. First, AS-tDR-007333 binds and interacts with heat shock protein family B member 1 (HSPB1), enhancing **histone H3 lysine 4 monomethylation (**H3K4me1) and **histone H3 lysine 27 acetylation (**H3K27ac) at the MED29 promoter to activate MED29 expression. Second, AS-tDR-007333 stimulates transcription factor ETS domain-containing protein Elk-4 (ELK4) expression, which binds to the MED29 promoter and increases its transcription [76]. Additionally, tsRNA-07804 is significantly upregulated in vitamin D-treated H1299 cells. Subsequent studies revealed that vitamin D-mediated tsRNA-07804 triggers mitochondrial dysfunction by targeting CRK-like proto-oncogene protein (CRKL), thereby inhibiting non-small cell lung cancer progression [77].

4) Other solid tumors:

In breast cancer (BC), tsRNA-26576 promotes proliferation and migration of MDA-MB-231 cells while inhibiting apoptosis [78]. In pancreatic cancer (PC), tiRNA-Val-CAC-2 enhances pancreatic cancer cell migration and invasion by binding to the RNA-binding protein **far upstream element-binding protein 1 (**FUBP1), increasing its stability, and activating c-MYC transcription [79].

#### 3.1.2 Hematologic malignancies

The role of tsRNAs in hematologic malignancies is often associated with abnormal proliferation and differentiation disorders of hematopoietic stem cells. In acute myeloid leukemia (AML) patients, the Methyltransferase 1/WD repeat domain 4 (METTL1/WDR4) complex catalyzes m7G modification of tRNA, enhancing translation efficiency of specific mRNAs (e.g., HOXA9, MEIS1) to maintain AML cell proliferation and stemness. Knocking down METTL1 reduces tRNA stability, increases tsRNA production, decreases global translation efficiency, and significantly impairs AML cell proliferation [80]. In chronic lymphocytic leukemia (CLL), the tsRNAs ts-3676 and ts-4521 exhibit downregulated expression and mutations, suggesting that tsRNAs may possess oncogenic and/or tumor-suppressive functions in hematopoietic malignancies [81].

In lymphoma research, particularly regarding diffuse large B-cell lymphoma (DLBCL), scholars have focused on exploring tsRNAs. Related studies have successfully developed a model/classifier based on six tsRNAs (tsRNA-Leu-CAG, tsRNA-Pro-CGG, tsRNA-Gln-CTG, tsRNA-Cys-GCA, tsRNA-Leu-AAG, and tsRNA-Lys-CTT). This innovative tool not only enables early detection of the disease but also effectively monitors treatment response and predicts prognosis. When combined with the International Prognostic Index (IPI), this model significantly enhances the accuracy of clinical stratification, demonstrating great potential as a liquid biopsy target [82].

### 3.2 Neurological diseases

In neurological disorders, tRNAs primarily participate in neuronal stress responses, autophagy regulation, and synaptic function maintenance. Their abnormal expression is closely associated with pathological processes in neurodegenerative diseases and cerebrovascular injury. Several tRNAs have been proposed as potential biomarkers for disease diagnosis.

#### 3.2.1 Neurodegenerative diseases

1) Parkinson's disease:

tRF-02514 promotes Parkinson's disease (PD) by inhibiting autophagy through targeting ATG5, thereby enhancing inflammatory cytokine release from microglia, inducing pyroptosis, and accelerating neuronal loss [83]. Additionally, nuclear-derived RGTTCRA-tRFs were found significantly elevated in PD, while mitochondrial-derived MT-tRFs were markedly reduced. Blood testing revealed that the RGTTCRA-tRFs/MT-tRFs ratio demonstrated an AUC of 0.86 for diagnosing early-stage PD, outperforming traditional clinical scoring (AUC = 0.73), suggesting its potential for therapeutic monitoring [84].

2) Alzheimer's disease:

Abnormal accumulation of glutamate tRNA fragments (tRF-Glu) in Alzheimer's disease (AD) patients' brains competitively binds mitochondrial leucine synthetase (LaRs2, a mitochondrial aminoacyl-tRNA synthetase involved in mitochondrial protein translation), disrupting leucine tRNA synthetization and mitochondrial protein translation. This leads to mitochondrial cristae structural damage and impaired glutamate synthesis. Clinical studies reveal that tRF-Glu levels in AD patients' cerebrospinal fluid positively correlate with tau pathological burden (r = 0.68, P < 0.001) and significantly negatively correlate with disease severity (MMSE score) [85]. Furthermore, elevating tRF-Ala-AGC-3-M8 levels to suppress **Ephrin type-A receptor 7** (EphA7) expression effectively blocked ERK1/2-p70S6K signaling in BV-2 and HT22 cells, thereby mitigating neuroinflammation and neuronal damage in AD [86].

#### 3.2.2 Cerebrovascular diseases

tRF-AspGTC promotes vascular smooth muscle cell (VSMC) phenotypic conversion and inflammatory responses by inhibiting Tripartite motif-containing protein 29 (TRIM29, a Protein Coding gene)-mediated galectin-3 ubiquitination, thereby activating the TLR4/MyD88/NF-κB signaling pathway and ultimately contributing to intracranial aneurysm (IA) formation [87].

### 3.3 Cardiovascular diseases

In cardiovascular diseases, tRNAs participate in the development of myocardial injury, hypertension, and atherosclerosis by regulating cardiomyocyte apoptosis, vascular endothelial function, and lipid metabolism balance. Some of these mechanisms offer novel insights for targeted therapies in cardiovascular diseases.

#### 3.3.1 Myocardial diseases

1) Myocardial infarction:

In myocardial infarction (MI), tRF-hc83 binds to a specific region of lncRNA MIAT, inhibiting MIAT's adsorption of VEGFA mRNA and thereby releasing MIAT's suppression of VEGFA translation. This promotes myocardial tissue angiogenesis and reduces infarct size [58]. Additionally, tRF5-22-SerGCT-1 may protect the heart from myocardial injury by targeting Mitogen- and stress-activated protein kinase 1 (MSK1) and regulating apoptosis through the MAPK pathway [88].

2) Cardiomyopathy:

In cardiomyopathy (CMP), 5′tiRNA-Gln-TTG-001 expression is significantly upregulated in acute myocarditis and positively correlates with the ratio of high-sensitivity cardiac troponin T to troponin T. This molecule activates the Chloride intracellular channel protein 4 (CLIC4, a membrane-associated ion channel protein regulating cellular inflammation and damage) gene by binding to its 3′ untranslated region, thereby exacerbating inflammatory damage to cardiomyocytes through increased CLIC4 expression [89].

#### 3.3.2 Vascular diseases

1) Hypertension:

Beyond affecting vascular physiology, Hypertension (HTN)'s regulatory role in the reproductive system is gaining attention, particularly regarding tsRNA modulation in sperm and transgenerational effects. HTN not only directly impacts spermatogenesis through hemodynamics and inflammatory injury but also potentially transmits pathological information to offspring by reshaping sperm tsRNA profiles. Taking tsRNA-00051 as an example, it acts as a competitive endogenous RNA (ceRNA) for miR-128-1-5p, jointly targeting the ArfGAP with GTPase domain, ankyrin repeat and PH domain 1 (AGAP1) gene to regulate angiogenesis and cell migration, thereby influencing spermatogenesis and function [90]. This study first links HTN with the epigenetic regulation of sperm tsRNAs, offering a new perspective on understanding HTN's impact on male reproductive health.

2) Atherosclerosis:

Atherosclerosis (AS) primarily manifests as lipid deposition, plaque formation, and intimal inflammation. Studies indicate that tRF-Gly-GCC promotes atherosclerotic plaque formation by regulating cellular adhesion, proliferation, and migration in human umbilical vein endothelial cells (HUVECs) and vascular smooth muscle cells (VSMCs) [91].

### 3.4 Metabolic diseases

In metabolic disorders, tRNAs primarily participate in regulating glucose and lipid metabolism as well as insulin sensitivity. Their functional abnormalities are closely associated with the development of metabolic disorders such as diabetes and non-alcoholic fatty liver disease, offering new perspectives for studying the mechanisms underlying metabolic diseases.

#### 3.4.1 Diabetes and its complications

Diabetes and its complications exhibit diverse types, with variations in pathogenesis, clinical manifestations, and the regulatory roles of tsRNAs. The following discussion will focus on type 2 diabetes, which is relatively common in clinical practice, as well as its typical complication-diabetic nephropathy. The specific mechanisms by which tsRNAs play a role in these conditions will also be explored.

1) Type 2 diabetes:

Studies indicate that in type 2 diabetes (T2D) patients, the expression level of mt-tRF-LeuTAA in mitochondria is regulated by the upstream mTORC1 pathway. By directly modulating electron transport chain function and oxidative phosphorylation efficiency, it maintains β-cell insulin secretion capacity. Abnormal expression may exacerbate mitochondrial dysfunction and insulin secretion defects, suggesting mt-tRFs could serve as novel biomarkers for diabetes diagnosis and intervention [92].

2) Diabetic nephropathy:

Notably, studies have identified differential tRF expression in the serum of patients with diabetic nephropathy (DN) [93]: specifically, three tRFs (tRF5-GluCTC, tRF5-AlaCGC, tRF5-ValCAC) are significantly upregulated, whereas another three tRFs (tRF5-GlyCCC, tRF3-GlyGCC, tRF3-IleAAT) exhibit downregulated expression. tRF3-IleAAT targets Zinc finger protein 281 (ZNF281), inhibiting ferroptosis and extracellular matrix (ECM) synthesis; its overexpression ameliorates renal injury [94]. Some tRFs also participate in multiple signaling pathways, influencing cellular damage [95,96].

#### 3.4.2 Non-alcoholic fatty liver disease

In non-alcoholic fatty liver disease (NAFLD) models, tRF-Val-CAC-005 binds to the 3′ untranslated region (3′UTR) of SMAD family member 7 (SMAD7) mRNA, reducing its mRNA stability and inhibiting translation, ultimately decreasing SMAD7 protein levels. The reduction in SMAD7 leads to sustained activation of TGF-β receptors, enhancing downstream Smad2/3 phosphorylation. This promotes the activation of hepatic stellate cells (HSCs) (transitioning from a quiescent state to a myofibroblastic phenotype) and upregulates the synthesis of extracellular matrix ECM components such as collagen (e.g., Col1A1) and fibronectin, thereby driving the onset and progression of liver fibrosis in NAFLD [97].

### 3.5 Infectious diseases

In infectious diseases, tsRNAs participate in the pathological processes of viral and bacterial infections by regulating host immune responses or directly acting on pathogen RNA. Their effects exhibit a “double-edged sword” characteristic—they may either suppress pathogen replication or promote pathogen survival.

#### 3.5.1 Viral infections

1) Hepatitis B:

Hepatitis B virus (HBV) infection induces oxidative stress, which in turn activates angiopoietin. This activation promotes the cleavage of tRNA into 5′ tRNA fragments; these 5′ tRNA fragments represent a major subcategory of small RNAs derived from tRNA. The abundance of these 5′ tRHs correlates significantly with viral infection status and stress levels, forming a crucial molecular link between viral infection and host stress responses [98]. This association is particularly pronounced in specific tsRNA subtypes, such as serum tsRNA-Gly and tsRNA-Glu. During active HBV replication, serum levels of these two tsRNAs are significantly elevated compared to healthy controls, whereas no marked increase is observed in patients during the inactive phase [99].

2) **COVID-19:**

As a major global public health crisis, COVID-19 (caused by the SARS-CoV-2 virus) has emerged as a critical context for investigating the crosstalk between tsRNAs and viral infection. This association has garnered extensive attention during the pandemic, with studies indicating that SARS-CoV-2 infection induces significant alterations in stress-related small RNA levels in patient blood, particularly 3′CCA tsRNAs derived from tRNA-Gly. Notably, these changes are highly correlated with levels of the inflammatory marker C-reactive protein (CRP) [100].

#### 3.5.2 Bacterial infections

Changes in tsRNA expression during bacterial infections may serve as potential diagnostic markers, with supporting evidence from tuberculosis (TB) studies. High-throughput sequencing analysis of tsRNA expression profiles in serum samples from TB patients and healthy individuals revealed that tsRNA-Gly–CCC–2, tsRNA-Gly-GCC-1, and tsRNA-Lys-CTT-2-M2 were significantly elevated in patient serum, correlating with pulmonary lesion severity and acid-fast bacilli grades. These markers hold promise for TB diagnosis and treatment monitoring [101].

### 3.6 Other diseases

Beyond the primary disease areas mentioned above, research on tsRNAs in autoimmune diseases and wound repair is gradually emerging. Although sample sizes remain small, these studies offer new directions for exploring the mechanisms of rare diseases.

1) Autoimmune diseases:

Significant upregulation of tRF-3009 expression was observed in peripheral blood mononuclear cells from systemic lupus erythematosus (SLE) patients. This suggests its potential involvement in IFN-α-induced metabolic regulation of oxidative phosphorylation in CD4**^+^**T cells, thereby influencing SLE's immune dysregulation mechanisms [102].

2) Trauma and repair:

Expression of tsRNA-23761 is markedly elevated in hypertrophic scar (HS) tissue. By establishing a ceRNA network involving tsRNA-miRNA-mRNA interactions, it regulates fibroblast proliferation and collagen synthesis, contributing to excessive scar tissue proliferation [103].

## 4 Summary and outlook

As an emerging class of non-coding RNAs, tsRNAs have evolved from “byproducts of tRNA degradation” to a key research focus. Their unique generation mechanism (cleavage of mature tRNAs or precursors by specific nucleases) and classification (two major subtypes: tRFs and tiRNAs) lay the foundation for functional characterization. Biologically, tsRNAs exert broad regulatory effects on cellular physiology and pathology by modulating protein translation, participating in epigenetic modifications, mediating intercellular signaling, and forming cross-regulatory networks with other non-coding RNAs.

In disease research, abnormal tsRNA expression is closely associated with a wide range of pathologies, including tumors, cardiovascular diseases, orthopedic disorders, and genetic diseases. Their mechanisms involve regulating core biological processes, and they hold potential as diagnostic biomarkers and therapeutic targets.

While the diagnostic potential of tsRNAs is promising, their clinical translation is hindered by the aforementioned challenges. Parallelly, the therapeutic exploitation of tsRNAs is advancing with promising strategies focusing on delivery systems and modified exosome-based technologies. For delivery, activated neutrophil membrane-coated nanoparticles (neu MCs) specifically engineered for tsRNA therapy encapsulate tRF-Gly-CCC. These nanoparticles leverage neutrophil-specific adhesion molecules to precisely target inflamed vascular sites and protect the tsRNA from enzymatic degradation; preclinical studies in aortic dissection/aneurysm models confirmed they maintain the contractile phenotype of vascular smooth muscle cells, suppress MMP secretion, and reduce inflammatory responses to halt pathological vascular remodeling [104]. Complementarily, cabbage-derived exosome-like nanoparticles (CELN) offer a cost-effective, biocompatible alternative designed to encapsulate small noncoding tsRNAs, which accumulate at injury sites in arterial restenosis models, suppress smooth muscle cell proliferation, and reduce neointimal thickening by targeting the PI3K/AKT pathway [105]. In terms of therapeutic applications, tsRNA-15797-modified BMSC-derived exosomes have emerged as a novel strategy for osteonecrosis of the femoral head (ONFH): by directly targeting and inhibiting LFNG expression, these exosomes significantly enhance angiogenesis and migration of human umbilical vein endothelial cells (HUVECs), with clinical sample analysis revealing reduced plasma exosomal tsRNA-15797 levels in ONFH patients, confirming its pathological relevance [106]. Additionally, ASO-tsRNA chimeric molecules improve *trans*-splicing efficiency by blocking competitive splice sites, as demonstrated in spinal muscular atrophy models to boost functional SMN protein expression, providing a versatile platform for genetic disease treatment [107]. Collectively, these advancements lay a solid foundation for the clinical translation of tsRNA-based therapies.

Despite significant advancements, tsRNA research still faces multiple challenges that hinder clinical translation:

(1) Challenges in biomarker validation: ① Sample stability variations across bodily fluids (e.g., impact of storage conditions, hemolysis on serum tsRNA abundance [108]); ② Lack of quantification standardization due to high sequence homology, diverse isoforms, and base modifications [109]; ③ Specificity issues caused by length similarity to miRNAs and heterogeneous naming conventions [109].
(2) Challenges in basic mechanism research: ① Unclear specific functions and downstream target molecules of most tsRNAs; ② Incomplete understanding of the complex co-regulatory networks between tsRNAs and other biomolecules; ③ Limited insights into the molecular basis of tissue-specific expression and the functional impact of tsRNA modifications (e.g., dynamic changes in diseases).
(3) Challenges in therapeutic translation: ① Need for optimized delivery systems to enhance targeting efficiency and reduce enzymatic degradation; ② Lack of unified preclinical research protocols for evaluating therapeutic efficacy; ③ Safety and long-term efficacy verification in clinical trials.

Future research may focus on three interconnected priorities: first, integrating multi-omics technologies to map tsRNA expression profiles and regulatory networks across diverse diseases; second, developing highly specific detection methods and targeted intervention tools for tsRNAs; third, advancing their clinical validation as diagnostic biomarkers and therapeutic targets, particularly in personalized medicine. With technological advances and deeper mechanistic understanding, tsRNAs hold promise to provide novel perspectives and strategies for elucidating disease mechanisms, diagnostics, and therapeutics.

## Funding

YJ was supported by the Major Project Funding (No. NSZD2025022) from Nanshan District Health Bureau of Shenzhen City, China.

## CRediT authorship contribution statement

**Yanting Jiang:** Writing – review & editing. **Shuyou Liu:** Writing – original draft. **Guolin Li:** Writing – review & editing. **Chengcheng Tang:** Data curation. **Yangyang Zhang:** Methodology. **Junjie Li:** Data curation. **Zhirong Tan:** Software. **Shimeng Zhang:** Data curation. **Zhiyou Chen:** Resources. **Shulong Li:** Resources.

## Declaration of competing interest

The authors declare that they have no known competing financial interests or personal relationships that could have appeared to influence the work reported in this paper.

## Data Availability

No data was used for the research described in the article.
