# Supplementary material for: Biological functions of tsRNAs and research advances in human disease
Source: Biochem Biophys Rep. 2026 Jan 6;45:102438. doi: 10.1016/j.bbrep.2025.102438 (PMC12808512; doi:10.1016/j.bbrep.2025.102438)
Supplement: Multimedia component 1 [file mmc1.docx]

Table S1: Representative tsRNAs, their Mechanisms of Action, and Corresponding References Across Major Disease Categories.

| Disease Category | | | Specific Disease | Representative tsRNA | Mechanism of Action | Reference |
| --- | --- | --- | --- | --- | --- | --- |
| Level 1 Category | Level 2 Subcategory | |  |  |  |  |
| **Neoplastic Diseases** | Solid Tumors | Level 3 Sub-  subcategory | HCC | tRNA-Glu/TTC | HCETSR (derived from tRNA-Glu/TTC) suppresses HCC malignant progression via regulating the SPTBN1/catenin axis. | [67] |
|  |  | Digestive System Tumors |  |  |  |  |
|  |  |  | HCC | tRF-3023 | Directly targets CDKN1A (p21) mRNA, accelerates G1-to-S phase transition, and promotes malignant  proliferation. | [68] |
|  |  |  | CRC | tsRNA-GlyGCC | Regulated by N(7)-methylguanosine modification; targets SPIB to activate JAK1/STAT6 pathway, enhancing 5-fluorouracil resistance. | [69] |
|  |  |  | CRC | tRF3008A | Inhibits FOXK1 (a positive regulator of Wnt/β-catenin pathway), suppressing cell proliferation and migration. | [70] |
|  |  |  | GC | tRF-19-3L7L73JD | Inhibits cell proliferation and migration, induces apoptosis, and causes G0/G1 phase arrest. | [71] |
|  |  |  | GC | tRF-23-Q99P9P9NDD | Binds 3'UTR of ACADSB, regulating lipid metabolism and ferroptosis to promote tumor progression. | [72] |
|  |  |  | GC | tRF-Tyr | Competitively binds hnRNPD, inhibiting its interaction with c-Myc 3'UTR and regulating c-Myc/Bcl2/Bax pathway to suppress progression. | [73] |
|  |  | Neurological Tumors | Glioma | tRF-22 | TRMT10A downregulation diminishes tRNA-argcct m1G9 modification and stability, upregulating tRF-22; tRF-22 represses MXD1 by 3'UTR binding, alleviating HIF1A transcriptional repression and promoting VM formation. | [74] |
|  |  |  | Glioma | tRNA-Leu-CAA | Correlates with poor survival; associated with IDH mutations, potentially targeting RBM43 and HOXA13 (overexpression linked to poor prognosis). | [75] |
|  |  | Respiratory Tumors | NSCLC | AS-tDR-007333 | Activates MED29 via two mechanisms: 1) Binds HSPB1 to enhance H3K4me1/H3K27ac at MED29 promoter; 2) Stimulates ELK4 expression, which binds MED29 promoter to promote transcription. | [76] |
|  |  |  | NSCLC | tsRNA-07804 | Induced by vitamin D; targets CRKL to trigger mitochondrial dysfunction, inhibiting tumor progression. | [77] |
|  |  | Other Solid Tumors | BC | tsRNA-26576 | Promotes proliferation and migration of MDA-MB-231 cells, inhibits apoptosis. | [78] |
|  |  |  | PC | tiRNA-Val-CAC-2 | Binds RNA-binding protein FUBP1 to increase its stability, activating c-MYC transcription and enhancing cell migration/invasion. | [79] |
|  | Hematologic Malignancies | | AML | m7G-modified tRNA-derived tsRNAs | METTL1/WDR4-mediated m7G modification enhances translation of HOXA9/MEIS1; knockdown of METTL1 increases tsRNA production, reduces global translation, and impairs AML cell proliferation. | [80] |
|  |  |  | CLL | ts-3676, ts-4521 | Downregulated with mutations; potential oncogenic/tumor-suppressive functions in hematopoietic malignancies. | [81] |
|  |  |  | DLBCL | Six tsRNAs (tsRNA-Leu-CA, tsRNA-Pro-CGG, tsRNA-Gln-CTG, tsRNA-Cys-GCA, tsRNA-Leu-AAG, tsRNA-Lys-CTT) | Developed as a classifier for early detection, treatment response monitoring, and prognosis prediction; improves clinical stratification accuracy when combined with IPI. | [82] |
| **Neurological Diseases** | Neurodegenerative Diseases | | PD | tRF-02514 | Targets ATG5 to inhibit autophagy, enhances microglial inflammatory cytokine release, induces pyroptosis, and accelerates neuronal loss. | [83] |
|  |  |  | PD | RGTTCRA-tRFs/MT-tRFs | Nuclear-derived RGTTCRA-tRFs elevated, mitochondrial MT-tRFs reduced; their ratio (AUC=0.86) aids early PD diagnosis and therapeutic monitoring. | [84] |
|  |  |  | AD | tRF-Glu | Competitively binds mitochondrial LaRs2, disrupting leucine tRNA synthetization and mitochondrial protein translation; correlates with tau burden (r=0.68) and negatively with MMSE score. | [85] |
|  |  |  | AD | tRFAla-AGC-3-M8 | Suppresses EphA7 expression, blocks ERK1/2-p70S6K signaling, and mitigates neuroinflammation and neuronal damage. | [86] |
|  | Cerebrovascular Diseases | | IA | tRF-AspGTC | Inhibits TRIM29-mediated galectin-3 ubiquitination, activates TLR4/MyD88/NF-κB pathway, and promotes VSMC phenotypic conversion and inflammation. | [87] |
| **Cardiovascular Diseases** | Myocardial Diseases | | MI | tRF-hc83 | Binds lncRNA MIAT, inhibits its adsorption of VEGFA mRNA, releases VEGFA translation suppression, promotes myocardial angiogenesis, and reduces infarct size. | [58] |
|  |  |  | MI | tRF5-22-SerGCT-1 | Targets MSK1, regulates apoptosis via MAPK pathway, and protects against myocardial injury. | [88] |
|  |  |  | CMP | 5'tiRNA-Gln-TTG-001 | Binds 3'UTR of CLIC4 gene to activate its expression, exacerbating cardiomyocyte inflammatory damage; positively correlates with hs-cTnT/TnT ratio. | [89] |
|  | Vascular Diseases | | HTN | tsRNA-00051 | Acts as ceRNA for miR-128-1-5p, jointly targets AGAP1, regulates angiogenesis and cell migration, and influences  spermatogenesis/function. | [90] |
|  |  |  | AS | tRF-Gly-GCC | Regulates adhesion, proliferation, and migration of HUVECs and VSMCs, promoting atherosclerotic plaque formation. | [91] |
| **Metabolic Diseases** | Diabetes and its Complications | | T2D | mt-tRF-LeuTAA | Regulated by mTORC1 pathway; modulates mitochondrial electron transport chain function and oxidative phosphorylation efficiency to maintain β-cell insulin secretion capacity. | [92] |
|  |  |  | DN | tRF5-GluCTC, tRF5-AlaCGC, tRF5-ValCAC | Significantly upregulated in serum; involved in cellular damage via multiple signaling pathways. | [93] |
|  |  |  | DN | tRF5-GlyCCC, tRF3-GlyGCC, tRF3-IleAAT | Significantly downregulated in serum; tRF3-IleAAT targets ZNF281, inhibits ferroptosis and ECM synthesis, and ameliorates renal injury when overexpressed. | 1. , |
|  | Non-Alcoholic Fatty Liver Disease | | NAFLD | tRF-Val-CAC-005 | Binds 3'UTR of SMAD7 mRNA, reduces its stability/translation, activates TGF-β receptor/Smad2/3 signaling, promotes HSC activation, and drives liver fibrosis. | [97] |
| **Infectious Diseases** | Viral Infections | | HBV | 5' tRHs | Induced by HBV-mediated oxidative stress and angiopoietin activation; correlates with viral infection status and stress levels. | [98] |
|  |  |  | HBV | tsRNA-Gly ,  tsRNA-Glu | During active HBV replication, serum levels of these two tsRNAs are significantly elevated compared to healthy controls, whereas no marked increase is observed in patients during the inactive phase. | [99] |
|  |  |  | COVID-19 | 3'CCA tsRNAs (tRNA-Gly-derived) | Induced by SARS-CoV-2 infection; changes in blood levels correlate with inflammatory marker CRP. | [100] |
|  | Bacterial Infections | | TB | tsRNA-Gly-CCC-2, tsRNA-Gly-GCC-1, tsRNA-Lys-CTT-2-M2 | Significantly elevated in serum; correlates with pulmonary lesion severity and acid-fast bacilli grades, potential diagnostic/treatment monitoring markers. | [101] |
| **Other Diseases** | Autoimmune Diseases | | SLE | tRF-3009 | Upregulated in PBMCs; potentially involved in IFN-α-induced oxidative phosphorylation metabolic regulation in CD4^+^T cells, influencing immune dysregulation. | [102] |
|  | Trauma and Repair | | HS | tsRNA-23761 | Upregulated in scar tissue; regulates fibroblast proliferation and collagen synthesis via tsRNA-miRNA-mRNA ceRNA network, contributing to excessive scar proliferation. | [103] |
